# Advancing Earth-Abundant CZTSSe Solar Cells: Recent Progress in Efficiency and Defect Engineering

**DOI:** 10.3390/nano15211617

**Published:** 2025-10-23

**Authors:** Yusuf Selim Ocak, Fatih Bayansal

**Affiliations:** 1 Department of Physics and Engineering Physics, Morgan State University, Baltimore, MD 21251, USA; 2 Department of Electrical and Computer Engineering, University of Connecticut, Storrs, CT 06269, USA; fatih.bayansal@uconn.edu; 3 Institute of Materials Science, University of Connecticut, Storrs, CT 06269, USA

**Keywords:** CZTSSe, kesterite, efficiency improvement, defect engineering, doping, annealing, secondary phases, band tailing, interface engineering

## Abstract

The earth-abundant, ecologically friendly structure of kesterite Cu_2_ZnSn(S,Se)_4_ (CZTSe) solar cells, with their advantageous optoelectronic characteristics, including a direct bandgap (1.0–1.5 eV) and a high optical absorption coefficient (>10^4^ cm^−1^), have made them a very promising member of thin-film photovoltaics. However, the path toward commercialization has been slowed down by restraint such as high open-circuit voltage deficits, deep-level defect states, and compositional inhomogeneities that lead to charge recombination and efficiency loss. Despite these obstacles, very recent advances in material processing and device engineering have revitalized this technology. Incorporating elements like Ge, Ag, and Li; optimizing interface properties; and introducing methods like hydrogen-assisted selenization have all contributed to raising device efficiencies by around 15%. This review discusses recent progress and evaluates how far CZTSSe has come and what remains to be done to realize its commercial promise.

## 1. Introduction

**Figure 1:**
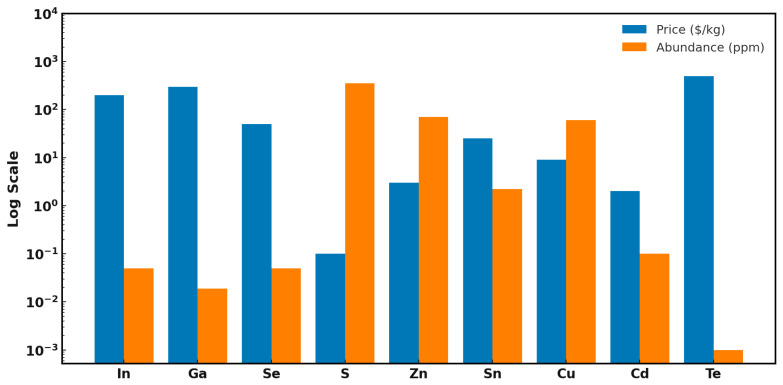
Log-scale bar chart showing material cost and earth abundance for elements in CdTe, CIGS, and CZTSSe solar cells.

Cu_2_ZnSn(S,Se)_4_ (CZTSSe)-based solar cells have garnered increasing attention in recent years as a sustainable and cost-effective alternative to conventional thin-film photovoltaic technologies [1,2,3,4,5,6,7]. The use of earth-abundant and low-toxicity elements [8,9,10,11] and favorable optoelectronic properties—such as a tunable direct bandgap (ranging from approximately 1.0 eV to 1.5 eV, or even up to 1.9 eV by alloying) [12] and strong light absorption (with coefficients typically exceeding 10^4^ cm^−1^) in CZTSSe solar cells—make them a compelling choice in the global push toward cleaner energy solutions and addressing climate change. As shown in Figure 1, CZTSSe combines high elemental abundance with low material cost, making it favorable compared to CdTe and CIGS.

Although the efficiency of CZTSSe-based solar cells could not increase over 12.6% power conversion efficiency (PCE) for a long time [9], in recent years, incremental progress has been reported [6,13,14,15], with certified PCEs for CZTSSe devices having recently approached 15% efficiency. This significant advancement, particularly after a decade of stagnation in performance, has renewed optimism about their long-term potential in addressing both energy demand and climate change justification goals.

Beyond efficiency metrics, the commercialization of thin-film photovoltaic technologies including CZTSSe critically depends on mass-production cost, scalability to large-area substrates, and long-term reliability of the produced modules [16,17]. In this context, the compatibility of CZTSSe with both rigid and flexible substrates provides additional application opportunities in lightweight, portable, and building-integrated photovoltaics [18,19].

**Figure 2:**
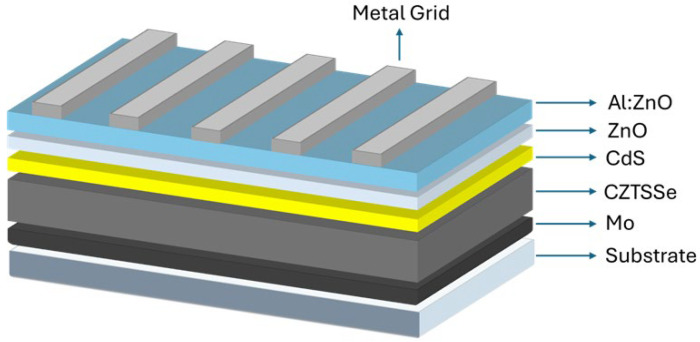
Schematic diagram of CZTSSe solar cell structure.

Despite recent progress, CZTSSe solar cells still fall behind well-known technologies like CIGS and CdTe, particularly in terms of open-circuit voltage (*V*_OC_) and fill factor (FF) [20,21]. These performance limitations largely originate from intrinsic material-related challenges. Defects including cation disorder (especially Cu_Zn_ antisite defects), deep-level Sn-related defects, and complex defect clusters are known to increase non-radiative recombination and band tailing, which limits the device efficiency [22,23]. Furthermore, the narrow phase stability range of kesterite materials cause formation of unwanted secondary phases like ZnSe, Cu_2_SnSe_3_, and SnSe_2_, which can degrade device performance by introducing recombination centers or impeding charge transport [24,25,26]. Other major concerns such as suboptimal band alignment and high defect densities between the absorber and buffer interface often lead to significant recombination losses. Similarly, reactions at the back contact (with Mo) can result in the formation of resistive molybdenum chalcogenides, which limit carrier extraction and reduce FF [27]. The basic device architecture of CZTSSe solar cells is illustrated in Figure 2, showing the layered structure from front contact to back contact.

To address these issues, a variety of strategies have been performed by researchers. While some of the studies concentrated on methods to obtain high-quality absorber layers including sol–gel methods, electrochemical deposition and physical vapor deposition techniques [5,28,29,30,31], many of them focused on doping strategies [15,32,33,34,35]. Cation substitutions including Ga, Ge, Ag, and Li have shown potential for reductions in recombination, improvements in crystallinity, and tuning in electronic properties [23,32,33,36,37]. It has also been showed that tailored bandgap engineering like S/Se or Ge/Ag alloying enables graded absorber profiles that enhance carrier collection [33,38]. Interface optimization by exploring alternative buffer materials and employing targeted annealing treatments has also helped relieve interface-related losses [39,40,41]. Additionally, advancements in processing techniques, including hydrogen-assisted selenization and multi-step annealing, are enabling better control over film composition and morphology [42,43,44].

A wide range of emerging photovoltaic technologies including halide perovskites, antimony selenide (Sb_2_Se_3_), dye-sensitized solar cells (DSSCs), and low-dimensional materials such as 1D nanowires and 2D layered semiconductors have demonstrated exceptional optoelectronic properties and rapid efficiency improvements [45,46,47,48,49,50,51,52]. However, despite their promising performance, many of these systems suffer from intrinsic challenges such as long-term instability, toxicity concerns, or limited scalability due to complex fabrication processes and low material abundance [45,46,47]. In contrast, earth-abundant kesterite compounds such as CZTSSe offer a unique combination of low toxicity, cost-effectiveness, and compositional tunability, making them strong candidates for sustainable photovoltaics [18,53]. Although several comprehensive reviews on CZTSSe solar cells have been published in recent years, most of them mainly focus on early developments or on a single topic such as post-annealing or alkali doping. In contrast, the present review provides an updated and integrated analysis of progress between 2021 and 2025, emphasizing recent defect passivation, interface engineering, and co-doping strategies together with their quantitative efficiency impact. This concise and critical perspective differentiates the present work from earlier general reviews. This review aims to bridge the gap between fundamental defect chemistry and practical device performance by systematically exploring defect engineering strategies alongside emerging commercialization pathways, such as flexible substrates, graded absorbers, and scalable annealing processes [54,55,56].

**Figure 3:**
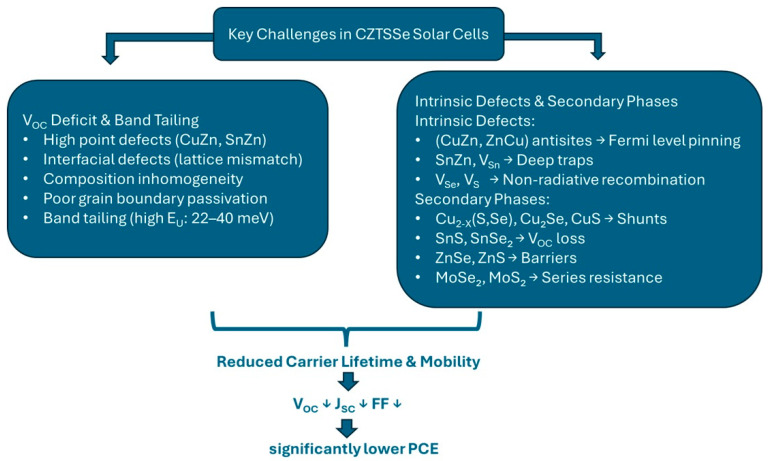
Schematic representation of the key performance-limiting factors in CZTSSe solar cells. Arrows indicate increase (↑) or decrease (↓) in the corresponding parameters.

Key performance-limiting factors are summarized schematically in Figure 3. In addition, unresolved challenges such as atomic-level defect characterization, band tailing reduction, and the development of cadmium-free buffer layers remain critical for future progress and are outlined later in this work.

To guide the reader, this article is organized as follows:

- Section 2 presents the key challenges that limit CZTSSe solar cell efficiency, including Voc deficit, defect formation, and secondary phases.
- Section 3 reviews recent efficiency improvement strategies such as doping/alloying, interface engineering, and morphology control.
- Section 4 provides outlook and commercialization aspects, including cost, scalability, flexible substrates, and stability.
- Section 5 concludes with a critical summary and future research directions.

Finally, device stability, a decisive factor for real-world deployment and long-term reliability, is also discussed in the conclusions as an essential complement to efficiency-focused strategies.

## 2. Key Challenges Limiting CZTSe Solar Cell Efficiency

### 2.1. Open-Circuit Voltage (V_OC_) Deficit and Band Tailing in CZTSSe Solar Cells

Kesterite CZTSSe-based solar cells are recognized as promising low-cost and environmentally benign devices, yet a persistent and large open-circuit voltage (*V*_OC_) deficit remains the most critical performance bottleneck [57]. This deficiency is an essential issue that maintains CZTSSe devices from achieving higher PCEs, which often means that their values are significantly below the theoretical Shockley–Queisser (SQ) limit [58,59]. The V_OC_ deficit is predominantly driven by non-radiative recombination arising from intrinsic and extrinsic materials issues, including the following:

- Highly concentrated point defects and defect clusters in the CZTSSe absorber layer [36];
- Interfacial defects caused by lattice mismatches [60];
- Changes in electrostatic and band potential caused by local composition inhomogeneity [61];
- Poorly passivated grain boundaries that act as recombination sites [2].

And, tailing is one of the major intrinsic factors that strongly affects both the open-circuit voltage (*V*_OC_) and the overall power conversion efficiency of CZTSSe solar cells. It originates from structural and compositional disorder in the absorber layer, which creates localized electronic states near the valence and conduction band edges. When these localized states become dense enough, they merge into band tails or impurity bands, effectively narrowing the optical bandgap and promoting non-radiative recombination [62]. Experimentally, this effect is often observed as a photoluminescence (PL) emission peak that appears significantly below the optical bandgap energy [63].

**Figure 4:**
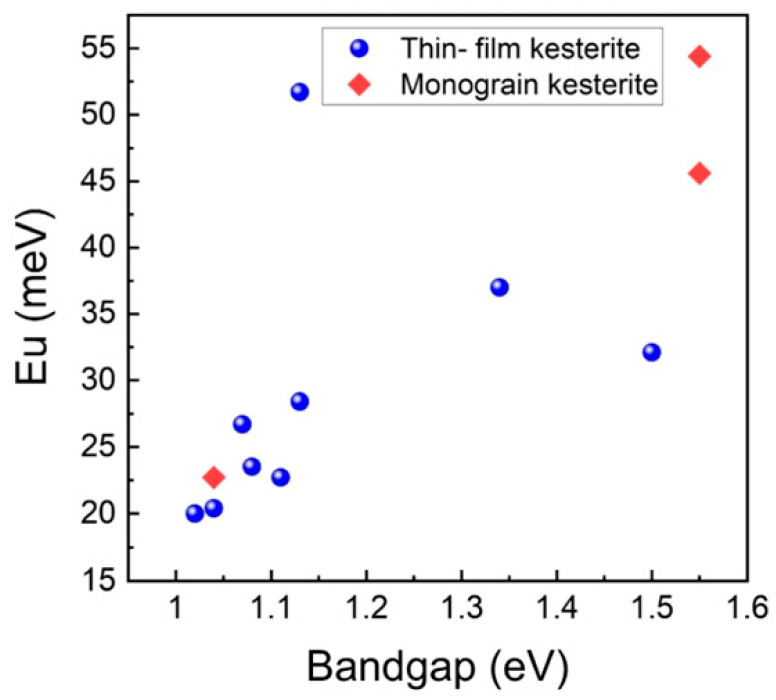
Relationship between Urbach energy (*E_U_*) and optical bandgap for kesterite absorbers. Reproduced from Ref. [18].

Urbach energy (*E_U_*) is a useful parameter for evaluating the degree of band tailing and structural disorder in kesterite semiconductors [64]. Lower *E_U_* values indicate sharper optical absorption edges and fewer localized tail states. As shown in Figure 4, *E_U_* gradually increases with the optical bandgap energy, suggesting that sulfur-rich kesterite compositions with wider bandgaps exhibit stronger band tailing and higher structural disorder than their selenium-rich counterparts. In addition, thin-film samples generally show slightly higher *E_U_* values than monograin materials, reflecting enhanced compositional fluctuations and electrostatic potential variations in polycrystalline absorbers. This trend confirms that stronger band tailing in wide-bandgap CZTSSe materials is a key factor responsible for the large *V_OC_* deficit commonly observed in kesterite solar cells.

Cu_Zn_ disorder is a big part of this problem because Cu_Zn_ antisite defects do not need much energy to form. Cu^+^ and Zn^2+^ have similar ionic radii, which makes it easier for cations to move randomly in the lattice. This causes big changes in the electrostatic potential and a lot of antisite defects [58,65]. The defect complex [2Cu_Zn_ + Sn_Zn_], especially, is a well-known cause of band tailing and potential fluctuations. At high concentrations, such defect clusters can form deep-level electron traps, significantly exacerbating non-radiative recombination [66]. Also, a high density of Cu_Zn_ defects can cause Fermi-level pinning, which weakens the band bending at the p–n heterojunction and increases interface recombination [36]. These defect–defect interactions amplify potential fluctuations and deepen band tails beyond the effect of individual antisites, suggesting that simple point-defect models may underestimate the true disorder in kesterite absorbers [67].

The combined effect of these defects is to shorten the lifetime and mobility of minority carriers, which makes it very hard to collect charges and leads to the big *V*_OC_ and PCE losses seen in CZTSSe solar cells [2,68].

### 2.2. Intrinsic Defects and Secondary Phases in CZTSSe Solar Cells

Kesterite CZTSSe-based solar cells are accepted as having great potential as low-cost and environmentally friendly photovoltaic devices, but they have some serious problems that make them less efficient. The CZTSSe phase has a very narrow thermodynamic stability window, which is one of the biggest issues because it makes it easier for different intrinsic defects and unwanted secondary phases to form [69]. These imperfections lower the performance of the device by acting as recombination centers or creating shunt paths [70,71].

#### 2.2.1. Intrinsic Defects

The complicated quaternary structure of CZTSSe has similar ionic radii for Cu^+^ and Zn^2+^ and ability of Sn to have more than one charge (Sn^2+^/Sn^4+^). This makes for a lot of defects and clusters that do not need a lot of energy to form, even in well-optimized films [72,73].

**Figure 5:**
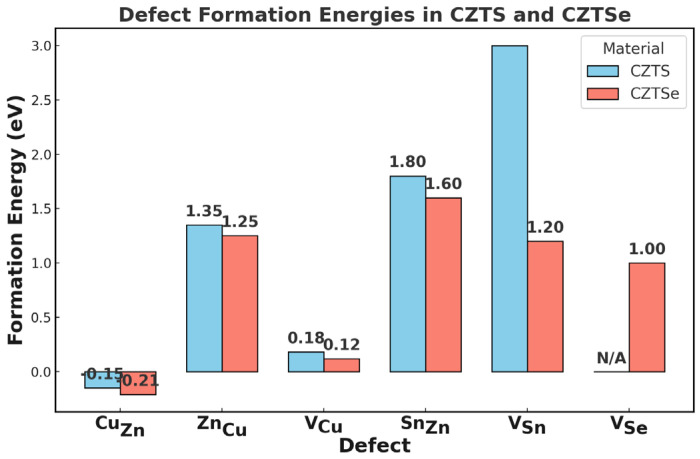
Calculated defect formation energies of dominant intrinsic defects in CZTS and CZTSe under Cu-poor/Zn-rich conditions. Blue bars represent CZTS, and red bars represent CZTSe. For CZTSSe alloys, values are expected to lie between the two end members depending on the S/Se ratio (x in S_1−x_Se_x_). Approximate values are shown for V_Sn_ and V_Se_. “N/A” indicates defects not relevant for the given composition [76].

Key Intrinsic Defects and Their Effects:

- Cu_Zn_ Disorder and Antisite Defects (Cu_Zn_, Zn_Cu_):

The very low formation energy of Cu_Zn_ and Zn_Cu_ antisite defects makes their equilibrium concentrations very high. The near-identical ionic radii of Cu^+^ (0.72 Å) and Zn^2+^ (0.74 Å) favor this disorder, causing random cation distribution and potential fluctuations [72,74]. As illustrated in Figure 5, the Cu_Zn_ antisite exhibits the lowest formation energy among the intrinsic defects. Although the [Cu_Zn_ + Zn_Cu_] antisite pair is sometimes regarded as “benign,” it is almost always present in CZTSSe and still contributes to electrostatic disorder and band tailing, especially near heterojunction interfaces [75].

- Sn-Related Defects (Sn_Zn_, V_Sn_) and Complexes:

Sn_Zn_ antisites and their associated complexes [Cu_Zn_ + Sn_Zn_] and [2Cu_Zn_ + Sn_Zn_] are widely recognized as deep-level recombination centers and strong drivers of band tailing [36,66]. These complexes create localized potential fluctuations that extend beyond the effect of single antisites. Reported Urbach energies (*E_U_*) above 30 meV for CZTSe confirm this stronger disorder, compared to ~15 meV for high-quality CIGS [61].

In addition, incomplete oxidation of Sn can stabilize Sn^2+^ states, producing lattice distortions and trap states with large carrier capture cross-sections. V_Sn_ vacancies are also deep-level traps and are often aggravated by Sn volatility or excessive Ge doping [77].

- Anion Vacancies (V_Se_, V_S_):

Sulfur and selenium vacancies (V_S_ and V_Se_) are deep donor defects that can trap carriers and enhance non-radiative recombination [78]. Incomplete sulfo-selenization is often linked to their formation.

- Other Defects (V_Cu_):

Copper vacancies (V_C*u*_) naturally act as shallow acceptors and may aid in p-type conductivity, especially when Cu_Zn_ defects are suppressed [36,72]. However, when combined with deep-level defects such as Sn_Zn_ or V_Sn_, overall carrier lifetimes and mobility decrease sharply, leading to poor charge collection and low PCE [61,66,77].

In summary, intrinsic defects, particularly Sn-related complexes and anion vacancies, are among the primary bottlenecks for *V_OC_* and fill factor in CZTSSe devices. Advanced in situ defect characterization techniques, such as synchrotron-based spectroscopy or deep-level transient methods, are still needed to fully understand and mitigate their impact.

Beyond defect energetics, their formation kinetics also play a crucial role in determining absorber quality. First-principles calculations indicate that Cu and Zn ions have very low migration barriers (≈0.2–0.4 eV), which explains the high probability of antisite disorder during high-temperature processing [79,80]. In contrast, Sn-related defects such as Sn_Zn_ exhibit higher formation barriers but, once present, form stable complexes that strongly affect recombination dynamics [36,81]. Defect–defect interactions further exacerbate disorder: clusters such as [2Cu_Zn_ + Sn_Zn_] are energetically favorable, acting as deep-level traps and amplifying band tailing beyond the effect of isolated antisites [82,83,84]. These insights underline the importance of kinetic control via optimized annealing atmospheres, slower cooling rates, or diffusion barriers, in addition to purely thermodynamic considerations [82,85,86].

#### 2.2.2. Secondary Phases

**Figure 6:**
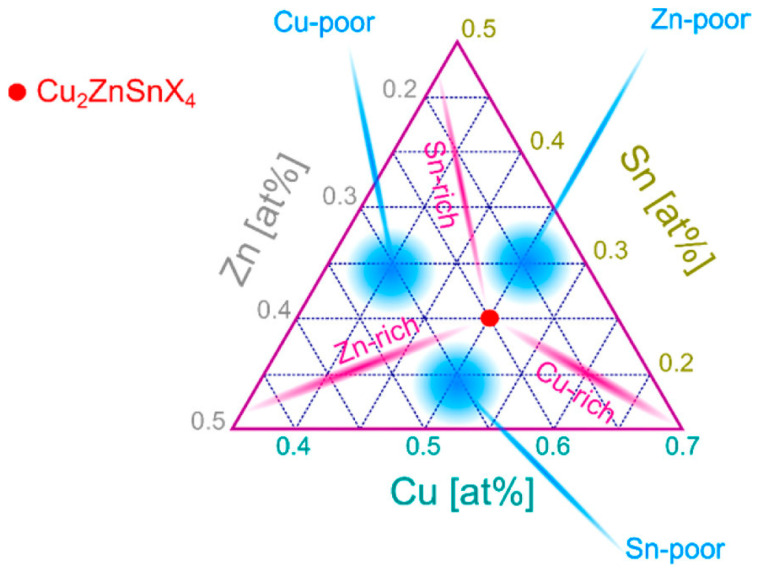
Cu–Zn–Sn–(S,Se) compositional phase diagram illustrating the stability window of kesterite and the formation conditions of secondary phases in CZTSSe thin films. The associated effects on device performance are discussed in the text. Reproduced with permission from [95].

Because CZTSSe is not very stable in its phase, it is likely to form secondary phases during synthesis. These phases can form if the stoichiometry is off, the precursors change, or the selenization or sulfurization is performed incorrectly. Figure 6 illustrates the compositional conditions that favor the formation of secondary phases, whose impacts on device performance are described in the following text. Common secondary phases and their effects can be listed as given below.

- Cu–S/Se Phases (Cu_2−x_(S,Se), Cu_2_Se, CuS):

These phases that conduct electricity very well are common when there is a lot of copper or when something breaks down. They can make shunt paths which make devices work a lot worse [87]. However, some reports suggest that very thin Cu_2_S interlayers may help stabilize interfaces if carefully controlled [88].

- Sn–S/Se Phases (SnSe, SnSe_2_, SnS, SnS_2_):

SnSe_2_ can reduce shunt resistance to some extent, but SnS negatively impacts Voc due to its narrower bandgap and its indication of Sn loss [89]. These phases are thermally unstable and often decompose at elevated temperatures, worsening long-term device reliability [90,91,92].

- Zn–S/Se Phases (ZnSe, ZnS):

These phases have a wide bandgap and are often found at the front and back of Zn-rich films. ZnSe regions near the back contact act as insulating barriers that reduce the effective absorber volume, thereby hindering carrier transport and decreasing Jsc and FF. While an ultrathin ZnSe layer at the interface may act as a beneficial passivation barrier, thicker layers increase series resistance [93].

- Molybdenum Chalcogenides (MoSe_2_, MoS_2_):

These layers form when the Mo back contact comes into contact with chalcogen vapors. A thin MoSe_2_ layer (<10 nm) can improve adhesion and promote quasi-ohmic contact, but excessive thickness (>50 nm) raises series resistance and may even lead to delamination of the absorber [94].

- Ternary Phases (e.g., Cu_2_SnSe_3_)

These form under Zn-poor or Cu-rich conditions. With a bandgap of ~0.8 eV, they create strong recombination centers and lower *V_OC_* [93].

#### 2.2.3. How It Affects the Performance of the Whole Device

Because CZTSSe solar cells simultaneously host intrinsic defects and secondary phases, they exhibit persistent *V_OC_* deficits, low fill factors, and high recombination rates. These problems hinder charge transport, reduce J_SC_, and ultimately cap PCE. To move closer to the theoretical efficiency limits of ~30%, it is essential to perform the following:

- Apply strict stoichiometric control;
- Optimize annealing/selenization protocols;
- Develop effective defect passivation and secondary phase suppression strategies.

Only by integrating these approaches can CZTSSe reach performance levels comparable to commercial thin-film technologies.

## 3. Strategies for Efficiency Improvement

**Figure 7:**
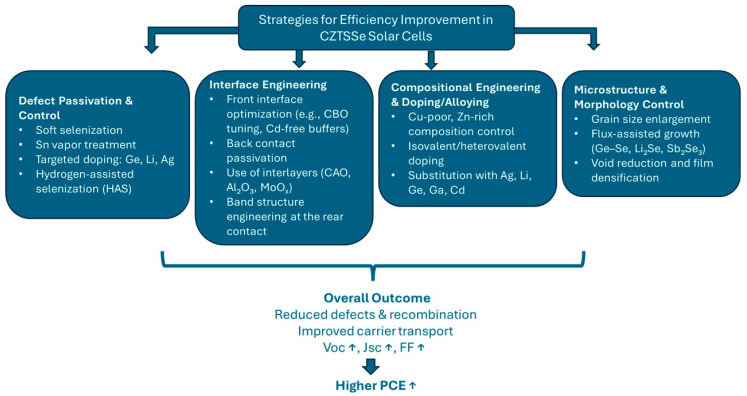
Schematic overview of strategies for improving the efficiency of CZTSSe solar cells. Arrows indicate increase (↑) or decrease (↓) in the corresponding parameters.

Recent improvement in CZTSSe solar cell efficiency has been realized using various synergistic approaches pointing to the challenges. Most important ones can be arranged as defect passivation and control, interface optimization, compositional engineering, and microstructural improvements. The following sections examine these strategies in detail, beginning with approaches to control bulk and interfacial defects, key contributors to the open-circuit voltage (*V*_OC_) deficit in CZTSSe solar cells. A schematic overview of these efficiency improvement strategies is provided in Figure 7, highlighting four major categories: defect passivation, interface optimization, compositional engineering, and microstructural improvements.

### 3.1. Defect Passivation and Control

In CZTSSe solar cells, defect passivation and control are essential to suppress non-radiative recombination, which is the main cause of the large open-circuit voltage (*V_OC_*) deficit and the limited power conversion efficiency (PCE). The complex crystal structure of the CZTSSe layer, which is characterized by common antisite defects including Cu_Zn_ and Sn_Zn_, defect clusters like [2Cu_Zn_ + Sn_Zn_], and secondary phases, results in electronic disorder and band tailing [22,26]. To overcome these limitations, a combination of chemical, structural, and interface engineering approaches is required. Recent advances have shown that targeted treatments and dopants can reduce defect formation, improve carrier transport, and enhance long-term device stability [96,97,98,99,100,101,102,103].

#### 3.1.1. Soft Selenization and Tin Vapor Treatment

Pre-selenization, also known as soft selenization, has proven effective in tailoring the local chemical environment during film growth. This treatment encourages the creation of fully oxidized Sn^4+^ species and harmless shallow acceptor defects like V_Cu_, thereby mitigating harmful defects such as Sn_Zn_, Cu_Zn_, and their complexes. It also helps to reduce the development of secondary phases during the annealing [104,105]. As a result, certified efficiencies up to 12.5% have been achieved in aqueous-processed CZTSSe devices, demonstrating the role of soft selenization in bulk defect passivation.

Similarly, supplying an excess of Sn vapor during annealing has been shown to stabilize the quaternary phase and prevent decomposition into secondary phases such as Cu_2−x_(S,Se) and Zn(S,Se). Sn vapor also inhibits Zn atoms from occupying Sn sites, thereby reducing Sn_Zn_ defect concentrations and improving *V_OC_* and fill factor (FF) [106,107].

Together, soft selenization and Sn vapor treatments improve stoichiometric balance, suppress deep-level traps, and enhance absorber quality, ultimately leading to better charge collection and higher device efficiency [108,109].

#### 3.1.2. Isovalent and Alkali Metal Doping

Germanium (Ge) Doping:

Among all examined dopants, the Ge atom has proven to be one of the most efficacious doping materials for regulating bulk defects and improving crystallinity. Ge^4+^ ions substitute Sn^4+^ sites, suppressing Sn^2+^ formation and reducing related deep-level recombination centers. This substitution reduces the density of Cu_Zn_ and Sn_Zn_ defects and can significantly decrease the integrated defect state density from 3.73 × 10^15^ cm^−3^ to 2.19 × 10^15^ cm^−3^ [110]. 

Ge doping also reduces the activation energy of Cu_Zn_-related defects (from ~157 to ~112 meV), decreases the Urbach energy (*E_U_*), and promotes grain growth, producing denser absorber layers with smoother morphology [110,111]. Additionally, Ge promotes grain growth, producing absorber layers that are denser and smoother **[110,111]**.

Lithium (Li) Doping:

Li doping, commonly introduced through Li_2_S or LiClO_4_, enhances p-type conductivity by forming shallow acceptor defects such as LiZn [112]. It can increase hole concentration by up to an order of magnitude, while reducing the density of defect clusters including [CuZn + SnZn] [113]. In addition, Li doping improves grain boundary passivation and moderates interface states, thereby enhancing band bending, Jsc, and overall carrier collection. Compared to Ag, Li provides more favorable conduction band alignment at the CZTSSe/CdS interface, enabling more efficient charge separation and transport [10].

Silver (Ag) Alloying:

Alloying or doping with silver atoms is also a promising method to decrease intrinsic defects and enhance crystallinity in CZTSSe absorbers [114]. Ag can substitute for Cu and, to a lesser extent, Sn or Zn. Replacing Cu with Ag increases the formation energy of CuZn antisites, thereby lowering their concentration. This reduces the density and depth of deep-level traps and mitigates defect clusters such as [2Cu_Zn_ + Sn_Zn_] [115]. At the same time, Ag promotes the formation of favorable complexes like [V_Cu_ + Zn_Cu_], improving overall defect energetics.

The incorporation of Ag into CZTSSe has been shown to enhance the optical quality of the absorber, as evidenced by sharper photoluminescence (PL) intensity and prolonged carrier lifetimes [116]. Reports indicate that Ag-alloyed films exhibit *E_U_* reductions from approximately 30 meV to around 27 meV, signifying diminished band tailing [114].

In addition, Ag passivation improves charge collection by reducing recombination at both bulk and interface levels, widening the space-charge region (SCR), and lowering the reverse saturation current density (*J*_0_). As a fluxing agent, Ag also promotes larger grain sizes and fewer grain boundaries, both of which are critical for minimizing recombination. Champion CZTSe devices with certified efficiencies approaching 14.9% have been achieved through the synergistic effects of Ag and Li doping [10].

Magnesium (Mg) Doping:

Recent work has introduced Mg^2+^ doping as a promising strategy to enhance cation ordering and suppress Cu_Zn_ antisite defects through a vacancy-mediated mechanism. The incorporation of Mg reduces deep-level traps and tail states, improving overall electronic order. Raman spectroscopy reveals a clear suppression of defect-related vibrational modes such as [V_Cu_ + Zn_Cu_] and a stronger A_1_(172) mode, with the A_1_(172)/[A_1_(172) + A_1_(194)] ratio increasing from 0.530 to 0.705. This structural refinement is directly correlated with a certified device efficiency of 15.3%, the highest reported for CZTSSe-based devices so far [14].

**Figure 8:**
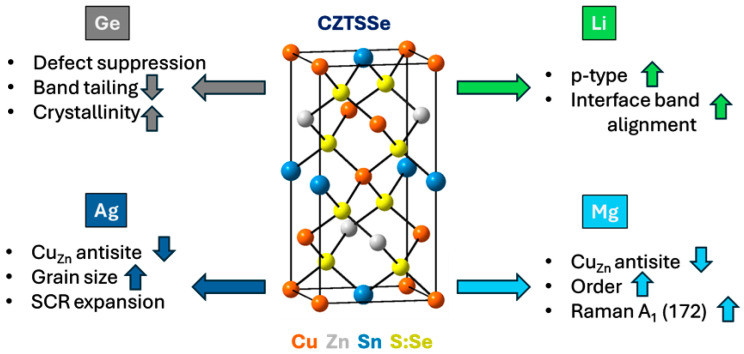
Schematic illustration of isovalent and alkali metal doping in CZTSSe absorbers. Ge substitutes Sn sites, suppressing Sn^2+^ formation and reducing band tailing. Li substitutes Zn, creating shallow acceptors and enhancing p-type conductivity and interface alignment. Ag substitutes Cu, raising the formation energy of Cu_Zn_ antisites, reducing recombination, and promoting grain growth. Mg substitutes Zn, suppressing antisite defects, improving cation ordering, and enhancing the Raman A_1_(172) mode. Arrows next to physical parameters (↑/↓) indicate increase or decrease, whereas large colored arrows represent the direction of dopant influence in the schematic.

As illustrated in Figure 8, the incorporation of Ge, Li, Ag, and Mg dopants into CZTSSe modifies the lattice at specific cation sites, suppressing detrimental defects, reducing band tailing, improving crystallinity, and ultimately enhancing device efficiency.

Although these dopants have led to notable improvements in Voc and efficiency, each strategy also involves specific trade-offs that must be carefully considered. For instance, Ge incorporation can promote grain growth but may induce Sn volatility when used in excess; Li diffusion is beneficial for passivation yet thermally unstable at high processing temperatures; Ag substitution improves crystallinity but may cause phase segregation at concentrations above a few atomic percent; and Mg doping can reduce deep defects but sometimes lowers carrier density. These examples illustrate that the benefits of doping are highly composition-dependent and require fine optimization to avoid secondary effects that counteract efficiency gains.

#### 3.1.3. Hydrogen-Assisted Selenization (HAS)

Hydrogen-assisted selenization (HAS) is a recently developed technique that enhances selenium incorporation by converting Se_8_ molecules into highly reactive H_2_Se through the action of H_2_ gas. This increases Se diffusion into the absorber; suppresses selenium vacancies (V_Se_), a major source of deep donor defects and carrier trapping; and improves stoichiometry and film homogeneity [117,118].

Admittance spectroscopy shows that HAS-treated films exhibit a reduction in average defect activation energy from 184 meV to 145 meV, indicating shallower defect levels. Device performance also improves, with J_SC_ increasing from 33.6 mA/cm^2^ to 36.1 mA/cm^2^, reflecting enhanced carrier collection efficiency [117]. Nanoscale X-ray fluorescence (nano-XRF) and X-ray beam-induced current (XBIC) mapping further confirm more uniform elemental distributions in HAS-treated absorbers, leading to better charge separation and transport.

**Figure 9:**
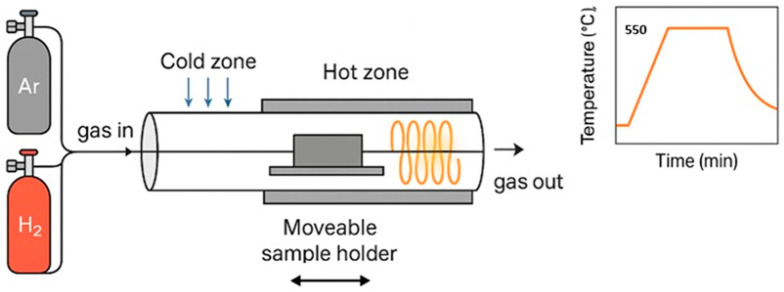
Schematic of the HAS process. H_2_ and Ar gases are introduced into a two-zone rapid thermal furnace. The Se precursor, initially located in the cold zone, reacts with H_2_ to form H_2_Se, which diffuses into the CZTSSe precursor positioned in the hot zone. This enhances selenium incorporation and suppresses anion vacancies, leading to improved absorber quality. Adapted from [117].

While HAS provides smoother surface morphology and better film quality, excessive Se diffusion must be avoided since it can negatively affect the CdS/CZTSe junction. The overall mechanism is illustrated in Figure 9, where Se_8_ is converted to H_2_Se and diffuses into the CZTSSe precursor, suppressing anion vacancies and improving absorber quality [117].

Together with soft-selenization [104], Sn vapor treatments, and dopant engineering (Ge, Li, and Ag), HAS has contributed to certified CZTSSe device efficiencies approaching 15%, highlighting its promise as part of a scalable and sustainable thin-film photovoltaic technology platform.

### 3.2. Interface Engineering

Interface engineering is one of the critical methods to improve the performance of kesterite CZTSSe solar cells. The efficiency of these devices is seriously influenced by interfacial recombination, improper band alignment, and non-ideal band bending, particularly at the heterojunction and back-contact interfaces. These factors influence many factors, including carrier extraction, *V*_OC_ reduction, and FF restriction. Therefore, precise control and optimization of both the front and back interfaces are needed for advancing CZTSSe photovoltaic performance [119].

#### 3.2.1. Front Interface Optimization (Absorber/Buffer Layer)

**Figure 10:**
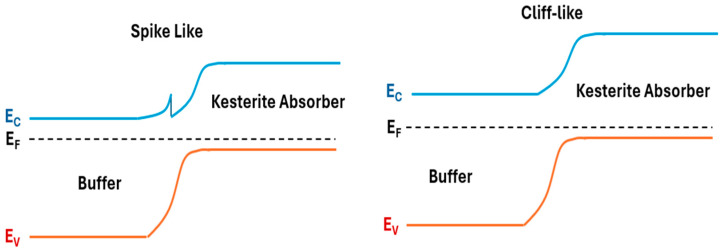
Schematics of spike-like (**left**) and cliff-like (**right**) band alignment at the buffer/kesterite heterojunction. Adapted from [1,124].

The front interface formed between the CZTSSe absorber, and the buffer layer (CdS) is a major site of non-radiative recombination, especially in sulfur-rich compositions. Optimizing this interface improves charge carrier separation and minimizes interfacial losses [120]. For efficient carrier transport, the conduction band offset (CBO) between the buffer and absorber layers must be within an optimal range. A spike-like CBO between 0 and 0.4 eV is considered ideal, since it acts like a barrier to back-injected electrons, thus suppressing recombination [121,122]. However, if the spikes exceed 0.4 eV, it may block electron flow. On the other hand, if the conduction level of the buffer band is lower than that of the absorber (a cliff-like alignment), it may cause recombination at the interface, which significantly reduces *V_OC_* and FF [122]. This effect is schematically illustrated in Figure 10, where cliff-like band alignment facilitates interface recombination, while a moderate spike-like offset acts as a barrier against back-injected electrons.

While CdS is still widely used, its environmental toxicity has motivated investigation of cadmium-free buffer layers including Zn(O,S), ZTO (ZnSnO), (Zn,Mg)O, In_2_S_3_, and TiO_2_. Zn(O,S) provides tunable band alignment by adjusting the S/O ratio, with positive CBOs (~0.4 eV) reported after surface treatments [112,113]. ZTO offers excellent transparency and a wide bandgap (3.2–3.6 eV), improves conduction band alignment, suppresses the “red-kink” in J–V curves, and allows for deeper valence band maximum (VBM) positioning. Efficiencies above 11% have been achieved in CZTSSe/ZTO structures [121]. (Zn,Mg)O, established in CIGS technology, enables band tuning and enhanced electron extraction. In_2_S_3_ can provide favorable spike alignment and promote diffusion into CZTSSe, creating shallow In_Sn_ acceptors. TiO_2_ and WS_2_ have also shown potential due to suitable electron affinities and benign interfaces [123].

In addition, extrinsic doping and interface treatments can further improve front-interface quality. Silver doping enhances the valence band maximum alignment by lowering the VBM of CZTSSe, thereby increasing band bending and reducing recombination [4]. Cd diffusion from the CdS buffer into the absorber layer creates Cd_Cu_ donors that form a buried n-type region, effectively acting as a homojunction and improving field-assisted separation while suppressing interfacial recombination [1,125]. Ga_Sn_ incorporation at grain boundaries introduces additional band bending, which blocks hole recombination channels [2]. Furthermore, Na–O enriched nanolayers such as ALD-deposited Al_2_O_3_ passivate interface defects and locally widened the bandgap, suppressing recombination and improving film uniformity [126].

#### 3.2.2. Optimization of the Back Interface (Absorber/Back Contact)

The back contact, usually molybdenum (Mo) is preferred, connects with the CZTSSe absorber through an interfacial Mo(S,Se)_2_ layer, which forms during selenization. This layer strongly influences hole extraction, series resistance, and device stability. A thin Mo(S,Se)_2_ layer (<10 nm) promotes quasi-ohmic contact and adhesion [7,127], whereas excessive growth (>50 nm) increases resistance, induces delamination, and degrades carrier collection [1]. To control this, several passivation strategies have been developed.

CuAlO_2_ (CAO) interlayers, Al_2_O_3_ nanopattern layers, and MoO_x_ passivation coatings are effective in managing the back interface. A thin CAO layer interposed between Mo and the absorber mitigates Se transport to Mo, hence preventing MoSe_2_ production [128]. It passivates interface states and functions as a hole-selective contact, facilitating grain expansion when combined with Ge doping [129]. Self-assembled Al_2_O_3_ nanoparticle islands can block elemental interdiffusion and restrict Mo sulfurization, while not entirely limiting charge transfer [130,131]. This facilitates the passivation of the back contact while maintaining its electrical conductivity. Similarly, oxidized molybdenum layers including MoO_3_ and MoO_2_ diminish the selenium reactivity of the back contact, so mitigating the excessive production of Mo(S,Se)_2_. Together, these passivation layers also reduce void formation and enhance adhesion [132].

**Figure 11:**
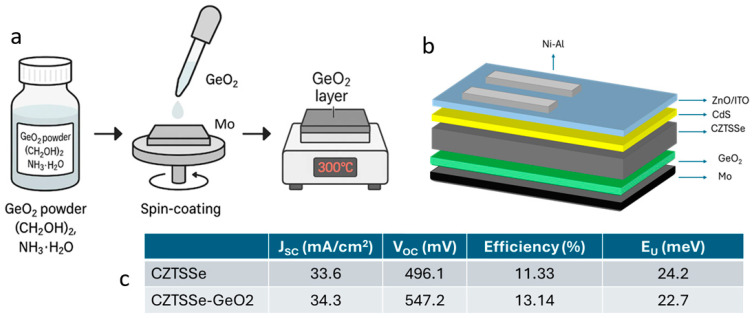
(**a**) Preparation and deposition process of the GeO_2_ layer on the Mo substrate via spin-coating and thermal treatment at 300 °C. (**b**) Schematic device structure of the CZTSSe solar cell incorporating the GeO_2_ layer. (**c**) Summary of photovoltaic parameters (Jsc, Voc, efficiency, and Urbach energy) for CZTSSe and CZTSSe–GeO_2_ devices. Adapted from [110].

Enhancements to the rear contact can substantially improve device performance. GeO_2_ or elemental Ge interlayers facilitate bidirectional diffusion during annealing. Upward Ge diffusion passivates bulk defects and reduces band tailing, while downward Ge diffusion increases the MoSe_2_ work function, acting as an electron reflector that boosts carrier separation and *V_OC_* [110]. These effects are clearly illustrated in Figure 11, which shows the device structure with a GeO_2_ layer, corresponding to *J*–*V* and EQE improvements, and the reduction in Urbach energy for Ge-incorporated absorbers.

Sn vapor treatments further stabilize the absorber during selenization, prevent decomposition into binary phases, and reduce Cu_Zn_ and Zn_Sn_ defects at the interface [75,133]. HAS also enhances Se diffusion, suppresses localized V_Se_ defects, improves elemental homogeneity, and increases *J_SC_* and carrier collection efficiency [117]. Moreover, applying thin Al_2_O_3_ layers under optimized processing conditions prevents Cu accumulation near the back contact, which can otherwise lead to compositional imbalance and defect-rich bottom regions [131].

Another approach is band structure engineering at the rear contact. Introducing p-type dopants into Mo, such as Na_3_PO_4_, raises its work function from ~4.7 eV to 5.6 eV, improving ohmic contact and optimizing band alignment, which enhances *V_OC_* [7]. At the same time, avoiding ZnSe secondary phase formation at the back contact is crucial, since ZnSe introduces unfavorable conduction and valence band offsets (CBO and VBO) that block carrier transport. Careful control of Zn concentration and refinement of selenization conditions can markedly suppress ZnSe formation [134].

Finally, a well-controlled three-step annealing process has shown promise in improving film quality [44]. This process begins and ends with low-selenium-pressure stages, separated by a high-temperature anneal in an inert atmosphere. Such a sequence limits MoSe_2_ thickness; reduces Sn loss and secondary phase formation; and produces a denser, more uniform absorber layer.

### 3.3. Compositional Engineering and Doping/Alloying

One of the best strategies to overcome the inherent performance limitations in CZTSSe solar cells is compositional engineering. By carefully adjusting elemental ratios and introducing suitable dopants, carrier transport can be improved, the band structure tuned, and harmful defects suppressed, all of which contribute to higher power conversion efficiencies. This approach is particularly important for CZTSSe due to its strong tendency to form antisite defects, its narrow thermodynamic stability window, and its structurally complex lattice. To move closer to theoretical limits, precise stoichiometric control and selective element substitution are essential.

A well-established guideline for achieving high device efficiency is to maintain a Cu-poor (Cu/(Zn + Sn) < 0.9) and Zn-rich (Zn/Sn > 1) compositions [3,9,135,136]. This chemical setting reduces the formation of deep-level defects such as Cu_Sn_, V_Sn_, and Sn_Zn_, which act as strong non-radiative recombination centers. It also suppresses secondary phases like Cu_2−x_(S,Se) and ZnSe, both more common under Cu-rich conditions, that can cause shunting and recombination losses. At the same time, a Cu-poor, Zn-rich composition increases p-type conductivity by promoting the formation of V_Cu_ shallow acceptor states. Another important benefit is the stabilization of fully oxidized Sn^4+^ species, which are far less detrimental to carrier lifetimes than Sn^2+^ [66].

Doping with isovalent or heterovalent elements allows for further control of defect populations, grain growth, and band alignment. For instance, substituting Ag for Cu has been shown to be particularly effective. Despite its similar ionic radius to Cu^+^, Ag^+^’s deeper 4d orbitals modify the electronic environment, raising the formation energy of Cu_Zn_ antisites and thus reducing band tailing and Fermi-level pinning [137]. Ag also promotes larger grains with fewer boundaries by acting as a fluxing agent [138]. It helps separate carriers and lessens recombination by slightly lowering the VBM, sharpening the band edges, and enlarging the depletion region [139]. These effects also improve band alignment at the CdS/CZTSSe interface. Ag doping has enabled efficiencies of about 13.4%, and when combined with lithium (Li), fill factors above 74% and PCEs close to 14.9% have been achieved [10].

Germanium (Ge) substitution on Sn sites also plays a critical role. Ge raises the conduction band minimum (CBM), widens the bandgap, and increases *V_OC_* [140]. It also suppresses the formation of deep-level defects such as Sn_Zn_ and Cu_Zn_ [15]. During selenization, Ge can form a Ge–Se liquid phase that promotes the growth of larger grains and reduces void density [38]. Ge has also been observed to migrate toward the back interface, forming a natural back surface field (BSF) that improves hole collection and reduces recombination losses [110]. When incorporated into MoSe_2_, Ge can increase its work function, further improving hole transport. Devices incorporating Ge have reached certified efficiencies of up to 14.67% [15].

Lithium (Li) doping, whether on Cu or Zn sites, is notable for its ability to increase hole concentration, sometimes by an order of magnitude, through the formation of shallow acceptor defects (LiZn) [141]. Li shifts the VBM downward and the CBM upward, optimizing conduction band offset at the CZTSSe/CdS interface. It also passivates defect clusters such as [Cu_Zn_ + Sn_Zn_], thereby suppressing band tailing [142,143]. When introduced Li_2_S or LiClO_4_, it enhances grain boundary passivation and crystallinity, extending minority carrier lifetimes. Li and Ag co-doping produces a strong synergistic effect: Ag improves morphology and suppresses antisite defects, while Li offsets the Ag-induced reduction in hole density. This combination has led to record efficiencies of nearly 14.91% [10].

Gallium (Ga) substitution for Zn or Sn offers another promising pathway. Ga reduces deep-level defects and increases band bending at grain boundaries by forming Ga_Sn_-related complexes [36]. It promotes more uniform grain sizes, reduces electronic inhomogeneity, and lowers the Urbach energy, indicating reduced electronic disorder [2]. Co-substitution on both Zn and Sn sites has been found to provide especially strong defect passivation. Devices produced with Ga doping via solution-based methods have achieved efficiencies above 12.1%, with significant improvements in *V*_OC_ [2]. The comparative influence of various dopants and co-doping combinations on CZTSSe device parameters is summarized in Table 1.

**Table 1:** Comparison of key dopants to CZTSSe and their effects.

Dopant	Substitution Target	Key Benefits	Max. Reported PCE (%)	Reference
Ag	Cu	Reduces Cu_Zn_ antisite defects, enlarges the depletion region width, improves grain growth, enhances band alignment, suppresses non-radiative recombination	13.38	[10]
Ge	Sn	Raises CBM, suppresses Sn_Zn_, improves morphology, back grading, reduces tailing	14.67	[15]
Li	Cu/Zn	Increases hole concentration, passivates GBs, improves alignment, synergistic with Ag	13.11	[10]
Ga	Zn/Sn	Suppresses deep defects, increases depletion width, reduces Urbach energy	12.12	[2]
Cd	Zn	Forms buried junction, increases hole density, improves lifetime	12.6	[144]
Ag + Li	Cu + Zn	Synergistic defect passivation and doping, highest FF and PCE	14.91	[10]
Ag + Ge	Cu + Sn	Improves front/back interface, grain growth, recombination suppression	8.46	[32]
Ge + Cd	Sn + Zn	Combines conductivity and band structure benefits	11.6	[145]

### 3.4. Microstructure and Morphology Control

The microstructure of the absorber layer plays an important role in kesterite solar cells for determining how efficiently charge carriers move and how effectively recombination can be suppressed. The best device performance is generally achieved when grains are large, densely packed, and contain few voids or gaps. A uniform morphology not only aids charge extraction but also supports defect passivation, which is essential for improving *V_OC_* and overall stability. Over the past decade, multiple strategies have been explored to achieve these structural goals in CZTSSe absorbers.

One of the most common ways is to take advantage of transient liquid phases during selenization. These low-melting intermediates can act as fluxes, helping small crystallites merge into larger grains and improving overall film densification. The Cu_2−x_Se liquid phase, for example, promotes both vertical and lateral growth, yielding a more compact absorber [146]. Germanium offers a different benefit: during the early stages of selenization, Ge–Se liquids form and spread quickly through the film, driving nucleation and lateral growth. This not only flattens the surface but also reduces void density, an effect especially evident in Ge-containing CZTGSe absorbers [15]. Sodium from soda-lime glass substrates may also enter the process through Na–Se liquids, assisting grain coalescence. Lithium-based fluxes, such as Li_2_Se, become active above about 350 °C and, when paired with silver co-doping, form Li_2_Se_x_ liquids that further promote grain growth [147]. Even SnSe_2_, when present in a molten form, can contribute by encouraging lateral expansion of CZTSe grains [148].

Co-selenization with antimony (Sb) provides another pathway. Adding Sb_2_Se_3_ during selenization enlarges grain size, increases packing density, and reduces pinholes. The fine-grained back-layer commonly seen near the Mo interface becomes thinner, while overall crystallinity improves. This effect is attributed to the formation of a Cu–Sb–Se liquid flux that enhances sintering, evens out elemental distribution, and reduces phase non-uniformities [149,150]. 

**Figure 12:**
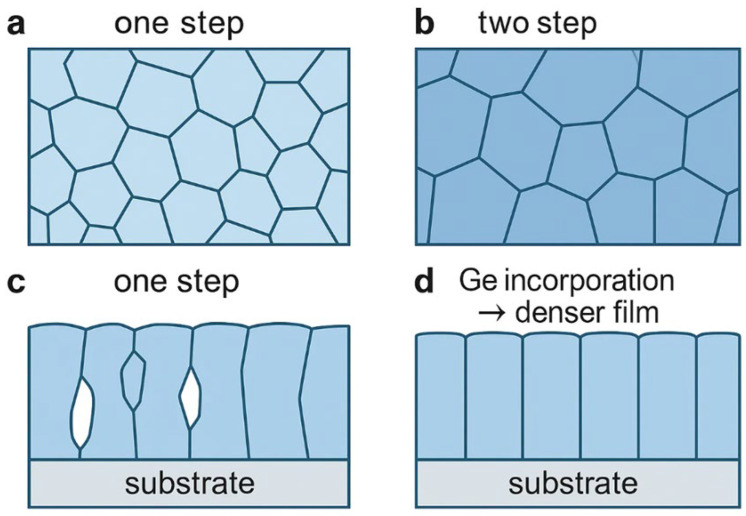
Schematic illustration of CZTSe thin films prepared by one-step (**a**,**c**) and two-step (**b**,**d**) selenization. One-step growth yields smaller grains and porous columns, while two-step selenization with Ge incorporation produces larger grains and denser films. Adapted in accordance with SEM observations [42].

The stacking sequence of precursor layers also strongly influences the final structure. For instance, Mo/Zn/Cu/Sn/Cu often yields a top-to-bottom growth pattern, producing large grains and minimizing nanoscale crystallites at the rear interface. This generally lowers series resistance and boosts fill factor [151]. By contrast, stacks such as Mo/Sn/Cu/Zn/Sn/Cu can result in incomplete crystallization near the back, likely due to the absence of the SnSe_2_-driven growth stage [152]. The density and texture of the initial precursor matter as well: loosely packed precursors allow deeper selenium penetration, while overly compact ones can hinder full reaction. Adjustments to sputtering power provide a way to fine-tune this, and in some cases, monolayer-like large grains can even be achieved without adding selenium during annealing [153]. These microstructural effects are clearly visible in Figure 12, which compares SEM images of CZTSe films produced by one-step and two-step selenization, demonstrating that the two-step process with Ge incorporation yields denser films and larger grains.

A number of additional parameters influence the final microstructure. Higher selenization temperatures tend to produce larger grains, although too much heat risks decomposition. Controlled two-step annealing starting at lower temperatures to incorporate selenium, followed by high-temperature crystallization, often provides the best balance [147]. Composition also plays a role; the familiar Cu-poor, Zn-rich regime not only suppresses unwanted secondary phases but also supports the growth of larger, more compact grains [66]. Magnesium doping, followed by selective surface etching, has been shown to promote Cu–Zn ordering and reduce antisite defects [154]. HAS improves selenium diffusion, smoothing the surface and minimizing Se-deficient regions [147]. Careful control of tin oxidation state can further activate shallow *V_Cu_* defects while suppressing deep-level centers such as Sn_Zn_ and Cu_Zn_ [75]. Finally, introducing a thin dielectric layer, such as CuAlO_2_, at the back contact has been shown to improve interfacial quality, which in turn supports the growth of a denser absorber with larger grains [110]. Despite these promising results, achieving uniform grain quality over large-area modules remains difficult, and the reproducibility of such microstructural improvements under industrial-scale processing conditions has yet to be fully demonstrated.

### 3.5. Commercialization Aspects

The commercialization of CZTSSe thin-film solar cells has received increasing interest due to their composition of earth-abundant, low-toxicity elements; suitable optoelectronic properties; and adaptability to low-cost, large-area manufacturing techniques. Unlike many competing thin-film technologies that rely on critical or toxic elements (e.g., Cd, In, and Ga), CZTSSe offers a sustainable materials platform for next-generation photovoltaics. However, successful commercialization requires significant progress in four key areas: cost competitiveness, process scalability, long-term device stability, and integration with flexible substrates.

#### 3.5.1. Cost Competitiveness

CZTSSe stands out as one of the most cost-effective absorber materials due to its use of inexpensive and abundant elements like copper, zinc, tin, sulfur, and selenium. This avoids the resource constraints and geopolitical risks associated with indium or gallium in CIGS and cadmium in CdTe [18,19].

Moreover, CZTSSe can be synthesized via low-temperature, solution-based techniques such as nanocrystal inks, spray pyrolysis, or electrodeposition, methods that drastically reduce capital and operational expenditures [18]. These methods enable roll-to-roll manufacturing, further enhancing the economics for large-scale deployment.

CZTSSe’s cost profile positions it particularly well for markets such as off-grid rural electrification, portable electronics, and indoor photovoltaics, where both affordability and environmental safety are paramount [19,155].

#### 3.5.2. Scalability and Manufacturability

Scalability is critical for CZTSSe to transition from laboratory-scale demonstration to industrial-scale production. Various scalable deposition techniques such as sputtering, thermal evaporation, spin coating, and spray pyrolysis have been successfully applied to CZTSSe absorber layers [156,157,158].

Roll-to-roll processing is particularly attractive due to its high throughput and low cost per watt. CZTSSe materials are compatible with continuous processing, enabling integration into large-area flexible modules [159,160]. Emerging research demonstrates successful implementation of precursors and low-temperature annealing processes in roll-to-roll systems [161,162].

Furthermore, the use of chemically stable and conductive back contacts such as Mo foils enhances process compatibility with flexible substrates [163,164].

#### 3.5.3. Device Stability

One of the main barriers to CZTSSe commercialization is its relatively low open-circuit voltage (*V_OC_*), largely attributed to bulk defects, antisite disorder, and interface recombination [160,165]. This *V_OC_* deficit limits theoretical power conversion efficiency (PCE) and is further exacerbated under thermal or illumination stress.

Alkali doping strategies, using Na, K, Li, or Ag, have shown promise in reducing defect density, increasing grain size, and stabilizing interfaces. Thermal and environmental stability has also been improved via passivation layers (e.g., Al_2_O_3_ and SiN_x_), back contact optimization, and barrier encapsulation against moisture and oxygen [163,166].

Experimental studies support these strategies, with one report demonstrating that CZTSSe devices maintained performance over 249 days of dry storage (~6000 h), indicating promising long-term stability under ambient conditions [167]. However, further improvements are needed to meet full IEC qualification standards for photovoltaic modules.

#### 3.5.4. Flexible Substrate Integration

CZTSSe solar cells are uniquely well-suited for integration with flexible substrates, enabling applications in wearables, aerospace, building facades, and indoor photovoltaic systems. Several studies have reported devices on PET, Mo foil, stainless steel, paper, and textile, with power conversion efficiencies over 11% in some cases [168,169,170].

**Figure 13:**
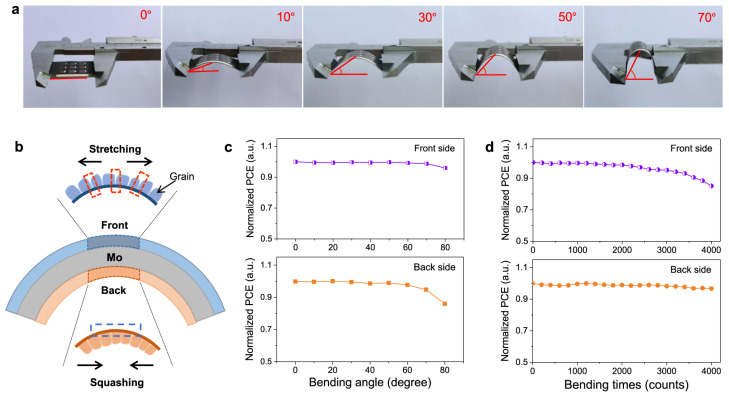
Example of bifacial flexible CZTSSe solar cells on Mo foil. (**a**) Photographs of devices bent at various angles (0–70°). (**b**) Schematic of stretching and squashing states on front and back sides. (**c**) Normalized PCE retention at different bending angles. (**d**) Evolution of PCE with repeated bending cycles (up to 4000), demonstrating >95% efficiency retention after 3000 cycles. Reproduced from [171], published under a Creative Commons CC BY license.

Innovative bifacial designs have been introduced, leveraging double-sided light collection to enhance energy yield under diffuse or multidirectional illumination conditions [171]. Additionally, the use of nanoporous or UV-treated substrates has demonstrated improved adhesion, bending durability, and mechanical resilience [163,172]. For example, Deng et al. demonstrated symmetrical bifacial flexible CZTSSe devices on Mo foil with front- and back-side efficiencies of 9.3% and 9.0%, stable operation under weak indoor lighting, and >95% retention of efficiency after 3000 bending cycles, underscoring the promise of flexible kesterite photovoltaics for wearable and indoor applications [171] (Figure 13).

Nonetheless, efficiencies on flexible substrates still lag behind those on rigid glass, primarily due to challenges in maintaining film crystallinity and controlling interfacial defects during high-temperature processing. Continued work on low-temperature fabrication and novel buffer layers is expected to close this performance gap.

In conclusion, CZTSSe solar cells exhibit strong potential for commercialization, owing to their low-cost composition, scalable processing, promising long-term stability strategies, and compatibility with flexible electronics. Although technical hurdles remain, especially in efficiency enhancement and defect passivation, ongoing progress across these four pillars significantly advances CZTSSe toward viable deployment in both conventional and emerging photovoltaic markets. Nevertheless, compared with mature thin-film technologies such as CIGS and CdTe, the gap in certified module efficiencies and industrial reproducibility is still substantial, highlighting that further breakthroughs in both absorber quality and large-scale manufacturing are required before CZTSSe can achieve true market competitiveness.

## 4. Advanced Characterization Techniques in CZTSSe Solar Cells

**Figure 14:**
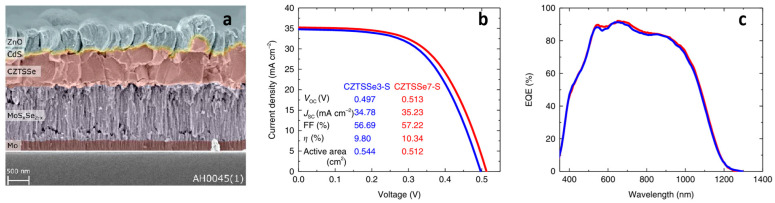
(**a**) SEM micrograph of the cross-section of a CZTSSe solar cell reproduced with permission [225], (**b**) *J*–*V* and (**c**) EQE curves of flexible CZTSSe3-S (blue) and CZTSSe7-S (red) solar cells reproduced from [159] published under a Creative Commons CC BY license.

Due to the complex defect chemistry and multiphase nature of CZTSSe thin films, a wide range of characterization tools are needed to examine performance-limiting elements, including antisite defects, band tailing, secondary phase formation, and interface quality [63,173]. In addition to standard structural and optical probes, advanced methods such as TEM, SEM/EDS, and in situ/operando spectroscopy are increasingly required to resolve nanoscale inhomogeneities and defect interactions at the atomic level [174,175]. The most popular methods and their diagnostic functions in CZTSSe research are compiled in Table 2. These tools facilitate future optimization by revealing hidden loss mechanisms, and track improvements from different doping and interface engineering strategies [176]. In addition, Table 3 and Table 4 provide reference peak positions for XRD and Raman spectroscopy, which are the two most important techniques in CZTSSe characterization, to help with phase identification and film quality assessment [177]. Moreover, electrical measurements such as *J*–*V* curves and quantum efficiency (QE) spectra remain indispensable to link structural observations with device performance, ensuring a comprehensive assessment of absorber quality and interface properties [173]. Representative SEM cross-sections and device-level performance (*J*–*V* and EQE) of flexible CZTSSe solar cells are shown in Figure 14, illustrating typical film morphology and photovoltaic response under optimized processing conditions.

**Table 2:** Common characterization techniques in CZTSSe solar cells.

Technique	Purpose in CZTSSe	Typical Target or Defect	Reference
XRD	Phase identification, crystallinity, alloying effects	CZTS(e), ZnSe, MoSe_2_, secondary phases	[178,179]
Raman Spectroscopy	Detecting secondary phases, Cu-Zn disorder, S/Se ratio	CTS, ZnS, SnS_2_, phase segregation	[11,180,181,182]
PL/TRPL	Evaluating band tailing and recombination activity	Cu_Zn_/Sn_Zn_-related tail states, minority carrier lifetime	[183,184,185]
DLCP	Bulk defect profiling	Cu_Zn_, Sn_Zn_ clusters, acceptor levels	[186,187]
SIMS	Elemental depth profiling, dopant distribution	Ge, Ag, Li gradients; Se and MoSe_2_ interface regions	[188,189]
EBIC/XBIC	Mapping charge collection efficiency	Spatial inhomogeneities, recombination centers	[190,191]
KPFM	Surface potential variation, work function mapping	Grain boundary barriers	[192,193]
SEM/TEM (with EDS/EELS)	Film morphology, grain size, thickness, nanoscale defect analysis	Grain boundaries, voids, secondary phases, antisite clusters	[93,194,195]
*J*–*V* & *QE* (Device-level tests)	Linking absorber quality to device performance	*V_OC_* deficit, FF losses, series resistance shunt resistance, carrier collection, spectral response	[196,197]
In situ/Operando spectroscopy	Real-time tracking of defect dynamics during annealing or illumination	Defect evolution, phase transitions, band tailing	[117,198,199]

Beyond the conventional probes listed in Table 2, several emerging techniques are becoming increasingly important for the CZTSSe field. Synchrotron-based X-ray absorption spectroscopy (XAS) and X-ray photoelectron spectroscopy (XPS) provide sensitive information on local bonding environments and oxidation states of cations, which are critical for identifying antisite defect complexes [200]. Atom probe tomography (APT) has been explored to directly visualize nanoscale phase segregation and dopant distribution in three dimensions [201]. Time-resolved terahertz spectroscopy (TRTS) and ultrafast pump–probe measurements offer insights into carrier dynamics and recombination pathways at sub-picosecond timescales, complementing time-resolved photoluminescence (TRPL) data [202,203]. In addition, operando Raman and photoluminescence mapping during annealing or light soaking have begun to reveal the dynamic evolution of secondary phases and band tail states under realistic processing and operating conditions [204,205,206]. These advanced approaches not only validate existing hypotheses about band tailing and defect clustering but also open new directions for correlating microscopic phenomena with macroscopic device limitations [207,208].

**Table 3:** XRD peak positions of CZTS(e) and secondary phases.

Phase	Major XRD Peaks (2θ, °)	Notes	Reference
CZTS (kesterite)	28.5, 47.3, 56.1	Often overlaps with ZnS and CTS; needs Raman confirmation	[209,210,211]
CZTSe	27.2, 45.2, 53.6	Slight shift due to larger Se radius	[212,213]
ZnS	28.5, 47.5	Overlaps with CZTS; cannot be unambiguously identified by XRD alone	[214,215]
Cu_2_SnS_3_ (CTS)	28.4, 47.3	Overlaps with CZTS; Raman needed to distinguish	[216,217]
MoSe_2_	13.4, 31.8, 38.3, 47.9, 56.1	Back contact phase; thickness and formation condition-dependent	[218]
ZnSe	27.2, 45.2, 53.6	Can appear near back contact in Zn-rich systems	[78,219]
SnS	22.0, 26.0, 27.5, 31.0, 44.8, 53.4	Orthorhombic α-SnS; peaks shift with morphology; overlaps partly with CZTS	[220]
SnS_2_	15.0, 28.2, 32.1, 50.1	Can overlap CZTS (28.5°); appears under Sn-rich conditions	[221]
Cu_2−x_Se	~27.0°, ~44.0–44.7°	Highly conductive; forms under Cu-rich conditions; creates shunt path	[87,222,223]
SnSe_2_	14.4, 31.15	Layered phase; common in Sn-rich or Se-rich films; affects *V*_OC_ stability	[224]
Cu_2_SnSe_3_ (CTSe)	28.44°, 32.96°, 47.31°, 56.13	Overlaps with CZTSe; requires Raman confirmation	[181,210]

**Table 4:** Raman peaks of secondary phases in CZTS(e) films.

Phase	Characteristic Raman Peaks (cm^−1^)	Notes	Reference
CZTS (kesterite)	~337, 287, 367	Strong peak at 337 cm^−1^; mode shift indicates S/Se ratio	[226,227]
CZTSe	~196, 174, 231	Main peak at 196 cm^−1^	[228,229]
ZnS	~274 (TO), ~351 (LO),	Cubic ZnS (sphalerite); TO and LO are reliable modes	[230]
CTS	~294, 354	Distinction from CZTS benefits from complementary techniques	[231]
SnS	68, 94, 162, 191, 219, 288	Orthorhombic α-SnS	[232]
SnS_2_	~205 (Eg), 312–315 (A_1_g), ~340 (A_2_u, weak)	Hexagonal phase; 205 and 312–315 cm^−1^ are dominant Raman modes, while the ~340 cm^−1^ mode is weak and rarely observed	[233]
MoSe_2_	~240 (A_1_g), ~285 (E_2_g^1^)	Appears when Mo reacts with Se during annealing	[234]
ZnSe	205, 250, 495	Zinc-blended ZnSe; overlaps with CZTSe, needs careful analysis	[235]
Cu_2_SnSe_3_ (CTSe)	178, 204, 231, 248	Often confused with CZTSe; Raman confirmation required	[236]
Cu_2−x_Se	260–270	Cu-rich phase; metallic character, introduces shunt paths	[237,238]

## 5. Conclusions and Outlook

CZTSSe solar cells have witnessed a significant efficiency leap in recent years, with reported PCEs now reaching 15.0%, which is a milestone that places kesterites among the most promising emerging thin-film photovoltaics. This progress is the result of a multifaceted research effort encompassing defect engineering, interface optimization, compositional tuning, and morphological control.

Defect engineering remains at the heart of performance enhancement. Controlling intrinsic point defects and defect clusters, especially Cu_Zn_, Sn_Zn_, and their complexes, is critical. Methods such as vacancy-assisted Cu–Zn ordering; Sn oxidation state control; and doping with elements like Ge, Ag, Li, and Mg have shown reliable success in reducing deep-level defects, improving p-type conductivity, and suppressing band tailing.

Interface engineering also plays a key role in reducing recombination losses. Strategies such as spike-type band alignment at the buffer/absorber interface, application of dielectric passivation layers, and heterojunction heat treatment (HJT) are very effective. At the back contact, inserting diffusion barriers and encouraging Ge to diffuse in both directions can make a noticeable difference. These steps help carriers move out more efficiently and also tend to stabilize the MoSe_2_ layer. In terms of composition, sticking with a Cu-poor, Zn-rich balance has worked consistently well for suppressing unwanted secondary phases. This effect becomes even stronger when the base composition is paired with dopants or alloying elements such as Sb, Ag, or Ge. Beyond defect control, these compositional tweaks let researchers nudge the bandgap into a more favorable range. The S/Se ratio also deserves careful attention; getting it right improves how the device matches the solar spectrum and helps the material hold up over time.

Bandgap grading offers another angle. A front surface enriched with Ag and a rear enriched with Ge can push carriers apart more effectively, reducing recombination losses. Even with these kinds of advances, CZTSSe still faces hurdles. The most stubborn are the voltage shortfall (*V_OC_* deficit) and fill factor limitations, both of which keep efficiencies well below the 23–24% already achieved by commercial CIGS and CdTe modules. The root causes are well-known band tailing in the absorber, recombination at grain boundaries or interfaces, and extra resistance within the device.

Another critical aspect of commercialization is device stability. Recent studies have shown that alkali metal and Ag doping, interface passivation, and optimized back-contact engineering can significantly improve thermal and operational stability. In some cases, CZTSSe devices have maintained over 90% of their initial efficiency after prolonged illumination or thermal stress tests, underscoring meaningful progress toward long-term durability. Nevertheless, stability under combined stress factors (heat, light, and humidity) remains an open challenge.

From an industrial and techno-economic perspective, the recent advances discussed here must also be evaluated in terms of scalability and long-term reliability. The compositional flexibility of CZTSSe enables its deposition on large-area and flexible substrates using low-cost sputtering or solution-based routes compatible with roll-to-roll processing. However, achieving device stability under IEC qualification tests and ensuring reproducibility at the module level remain key challenges. Continuous improvement in absorber uniformity, back-contact reliability, and encapsulation will therefore be essential for the large-scale commercialization of kesterite solar cells.

Looking ahead, targeted strategies will be necessary to overcome the remaining bottlenecks. These include developing in situ and operando techniques such as transmission electron microscopy and time-resolved spectroscopy to directly observe defect evolution, exploring multi-element co-doping schemes for enhanced defect passivation, and optimizing roll-to-roll or solution-based processes for industrial scalability. Furthermore, replacing CdS with cadmium-free buffer layers such as Zn(O,S), ZTO, or In_2_S_3_ will be essential for environmentally benign device architectures.

In particular, among the various approaches reviewed, co-doping strategies combining Ge with alkali elements such as Li or Na have shown the most reproducible improvements in open-circuit voltage and carrier lifetime, while interface engineering efforts that replace CdS with Zn(O,S) or In_2_S_3_ buffers demonstrate reduced recombination losses and enhanced stability. These trends indicate that the most promising future direction lies in synergistically optimizing both the absorber and interface, supported by improved defect control and large-area process uniformity.

Tackling these problems will require a closer look at what is happening at the microscopic level, especially how and where carriers recombine, and new ways to passivate those sites. It may also mean going beyond the usual dopants and exploring ways to shape the local chemical environment during growth. Combining methods is likely to be more effective than relying on any single approach. For instance, co-doping could be paired with interface treatments or phase boundary control. On the manufacturing side, simpler and more scalable routes will be important. Techniques like selenium-free annealing, sputtering quaternary alloys, or using solution-based processes such as electrodeposition all offer paths toward high-quality films without driving up costs or environmental impact.

Recent advances once again show the strategic importance of CZTSSe in the landscape of thin-film photovoltaics. The earth-abundant and non-toxic composition of CZTSSe, combined with the potential for low-cost, scalable manufacturing, places it as a compelling candidate for next-generation solar technologies. The recent progress in both efficiency and stability underlines that CZTSSe is not only a promising emerging absorber material but also a technology of lasting significance for the future of sustainable energy.

## Data Availability

The data is contained within the article.
